# Neuroprotective effects of chemical constituents from *Nicotiana tabacum* L. in Parkinson’s disease

**DOI:** 10.1007/s13659-025-00541-8

**Published:** 2025-10-01

**Authors:** Hao-Jing Zang, Xiao-Lin Bai, Xue-Yi Sui, Xiao-Rui Zhai, Yong-Cui Wang, Zhong-Quan Xin, Qiu-Yuan Yin, Xiao-Jiang Hao, Yue-Hu Wang, Xun Liao, Ying-Tong Di

**Affiliations:** 1 State Key Laboratory of Phytochemistry and Natural Medicines, Kunming Institute of Botany, Chinese Academy of Sciences, Kunming, 650201 China; 2 Chengdu Institute of Biology, Chinese Academy of Sciences, Chengdu, 610041 China; 3 School of Life Sciences, Yunnan University, Kunming, 650091 China; 4 University of Chinese Academy of Sciences, Beijing, 100049 China; 5 National Tobacco Genetic Engineering Research Centre, Yunnan Academy of Tobacco Agricultural Sciences, Kunming, 650021 China; 6 Research Unit of Chemical Biology of Natural Anti-Virus Products, Chinese Academy of Medical Sciences, Beijing, 100730 China; 7 Yunnan Characteristic Plant Extraction Laboratory, Kunming, 650201 China; 8 Yunnan Key Laboratory for Wild Plant Resources, Kunming Institute of Botany, Chinese Academy of Sciences, Kunming, 650201 China

**Keywords:** Parkinson's disease, neuroprotective effects, *Nicotiana tabacum* L, 21-norditerpenoid

## Abstract

Parkinson’s disease (PD), the second most common neurodegenerative disorder globally, arises from selective dopaminergic neuron degeneration. While current therapies address symptoms, disease-modifying agents remain an unmet need. Herein, we investigated *Nicotiana tabacum* L. (Solanaceae), a plant linked epidemiologically to reduced PD risk, as a source of multi-target neuroprotective compounds. From ultra-low nicotine (< 0.04%) tobacco leaves, we isolated 22 molecules, including a novel 21-norsesterterpenoid (Nicotiazanorpenoid A) and eight previously unreported compounds. Systematic evaluation revealed three synergistic neuroprotective mechanisms: (1) Antioxidant activity: Scopoletin (**3**) and isoferulic acid (**6**) showed significant radical scavenging capacity (ABTS assay; IC_50_ = 27.74, and 18.13 μM, respectively); (2) Neuronal protection: *cis*-11,14,17-Eicosatrienoic acid methyl ester (**14**) enhanced survival (93.94% vs. control) in 6-OHDA-induced PC12 cells, surpassing rasagiline (88.36% at equivalent concentrations); (3) MAO-B inhibition: five compounds displayed selective inhibition, with scopoletin (**3**) exhibiting highest potency (*K*_i_ = 20.7 μM). Notably, plant prostaglandins (**10/11**) were identified as competitive MAO-B inhibitors first time through molecular docking and 100-ns MD simulations, revealing stable binding at the FAD site (ΔG = − 10.42, and − 9.75 kcal/mol, respectively).

**Graphical Abstract:**

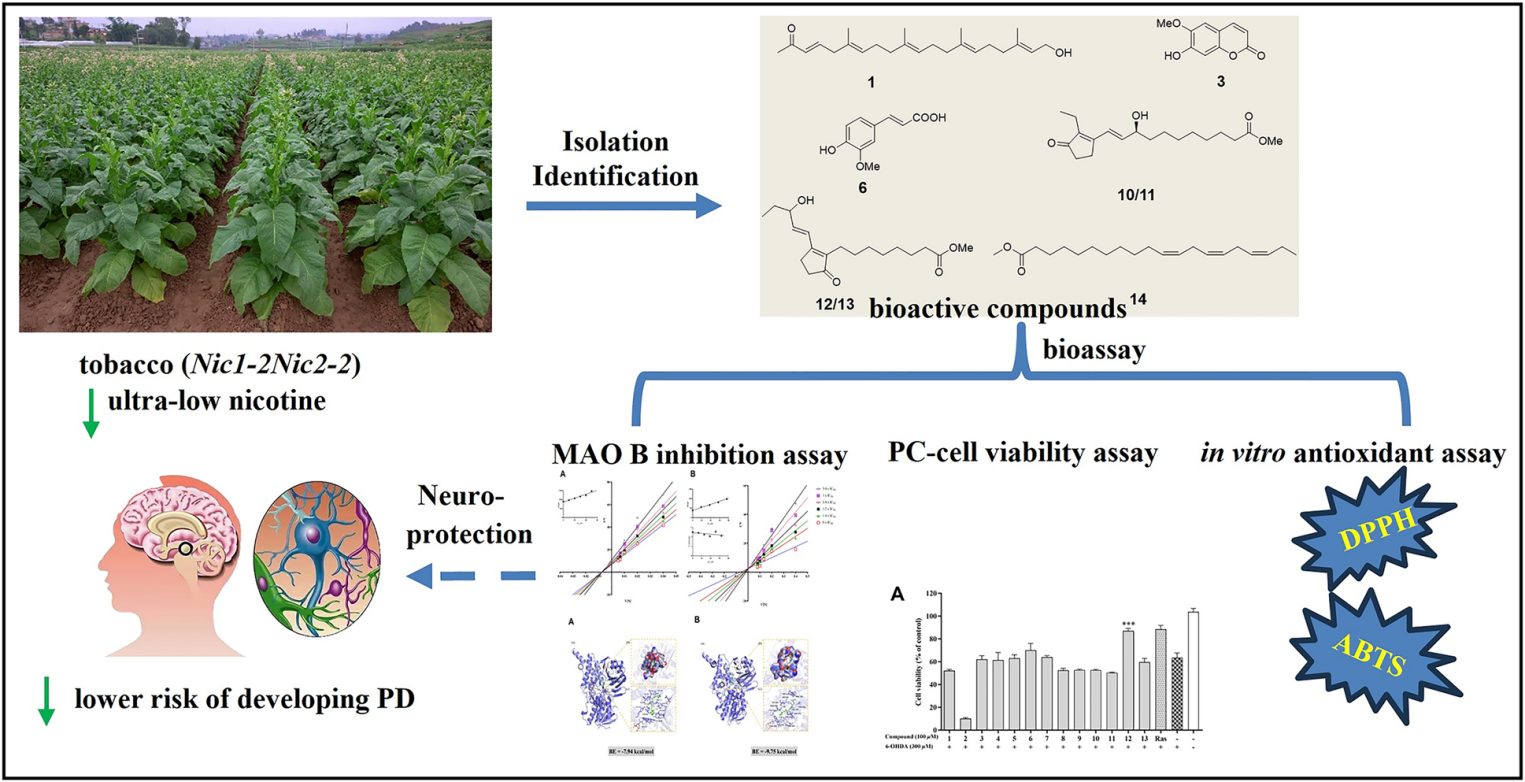

**Supplementary Information:**

The online version contains supplementary material available at 10.1007/s13659-025-00541-8.

## Introduction

Parkinson's disease (PD), a progressive neurodegenerative disorder affecting > 1% of individuals aged over 60 years worldwide [1], is characterized by the selective degeneration of dopaminergic neurons in the substantia nigra pars compacta. Notably, approximately 30% of neurons and 50–60% of axonal terminals are already lost at clinical diagnosis [2]. While current therapies (e.g., l-DOPA) provide symptomatic relief, no disease-modifying interventions exist to halt neurodegeneration [3]. This unmet need has driven extensive research into neuroprotective agents targeting PD’s multifactorial pathogenesis, including oxidative stress, mitochondrial dysfunction, and protein aggregation [4].

Intriguingly, epidemiological studies consistently report a reduced PD risk among tobacco users [5, 6], suggesting that *Nicotiana tabacum* L. (Solanaceae) may harbor bioactive compounds with neuroprotective properties. Although nicotine—the most studied tobacco alkaloid—failed to demonstrate clinical efficacy [7], the plant contains > 2500 identified metabolites [8], including terpenes, flavonoids, and cembranoid diterpenoids, some of which exhibit neuroprotective effects in vitro [9, 10]. These findings underscore the potential of non-nicotine constituents as novel therapeutic candidates.

Herein, we investigated the neuroprotective potential of low-nicotine (< 0.04%) *N. tabacum* extract [11] through a multi-target approach: (1) Inhibition of monoamine oxidase B (MAO-B), a key enzyme in dopamine catabolism; (2) Scavenging of reactive oxygen species (ROS) to mitigate oxidative stress; (3) Protection against 6-Hydroxydopamine (6-OHDA)-induced neurotoxicity in PC12 cells, a validated PD model. This study elucidates the phytochemical basis of tobacco’s putative neuroprotection and identifies promising leads for PD drug development.

## Results and discussion

### Compounds from leaves of the *N. tabacum*.

**Fig. 1:**
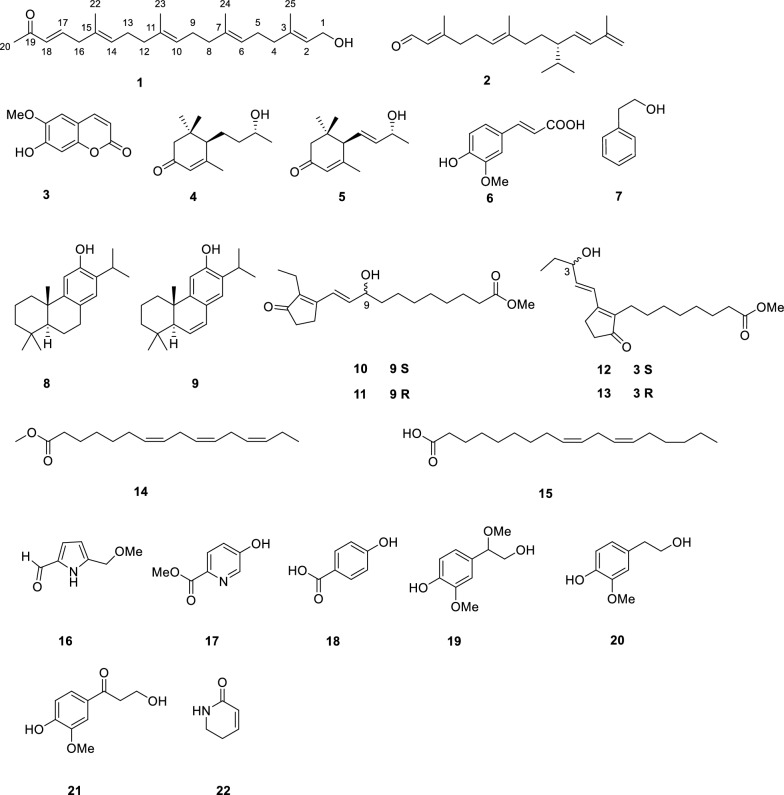
Compounds **1**–**22** from the leaves of *N. tabacum*

22 compounds (**1**–**22**) were isolated from *N. tabacum* leaves with ultra-low nicotine content, using solvent partitioning and diversified chromatography techniques, including normal, reverse, and molecular exclusion (Fig. 1). Among them, compound **1** is an undescribed 21-norsesterterpenoid, while compounds **8**–**14**, and **19** are reported from *N. tabacum* for the first time.

Nicotiazanorpenoid A (**1**) was acquired as a colorless oil. Its molecular formula of C_24_H_38_O_2_ was deduced by HRESIMS at *m/z* 381.2768 [M + Na]^+^ ion (calcd 381.2764), indicating six degrees of unsaturation. Its IR spectrum exhibited clear absorption bands. The one at 3441 cm^− 1^ indicated the existence of a hydroxyl group, while the band at 1637 cm^− 1^ suggested an α, β-unsaturated ketone system was present. The ^1^H-NMR data (Table 3) showed five quaternary methyl groups (*δ*_H_ 1.60 (s, 6H), 1.61 (s, 3H), 1.68 (s, 3H), and 2.25 (s, 3H)), one *O*-bearing CH_2_ group (*δ*_H_ 4.16, 2H, *J* = 6.5 Hz) and six olefinic protons (*δ*_H_ 5.42 (t, *J* = 7.0 Hz, H-2, 1H), 5.19 (t, *J* = 7.0 Hz, H-14, 1H), 5.11 (t, *J* = 6.5 Hz, H-10, 1H), 5.11 (t, *J* = 6.5 Hz, H-6, 1H), 6.60 (d,* J* = 16.0 Hz, H-18, 1H), 6.76 (dt,* J* = 16.0, 7.0 Hz, H-14, 1H). The ^13^C NMR and DEPT spectra (Table 3) displayed 24 carbon signals, including one carbonyl carbon (*δ*_C_ 198.9), four trisubstituted and one disubstituted C = C bonds (*δ*_C_ 146.7 (d), 139.9 (s), 135.5 (s), 134.8 (s), 132.2 (d), 131.4 (s), 127.6 (d), 124.7 (d), 124.0 (d), and 123.5 (d)), and seven sp^3^ CH_2_ (*δ*_C_ 42.7, 39.9, 39.7, 39.6, 26.9, 26.8, and 26.4). Besides the six degrees of unsaturation provided by five double bonds and one carbonyl group, the lack of any extra degrees of unsaturation indicates that compound **1** probably has a linear structure.

**Fig. 2:**
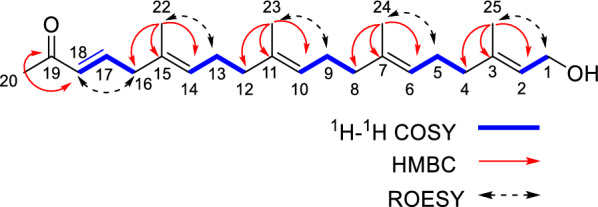
The ^1^H-^1^H COSY, HMBC and ROESY correlations of compound **1**

**Fig. 3:**
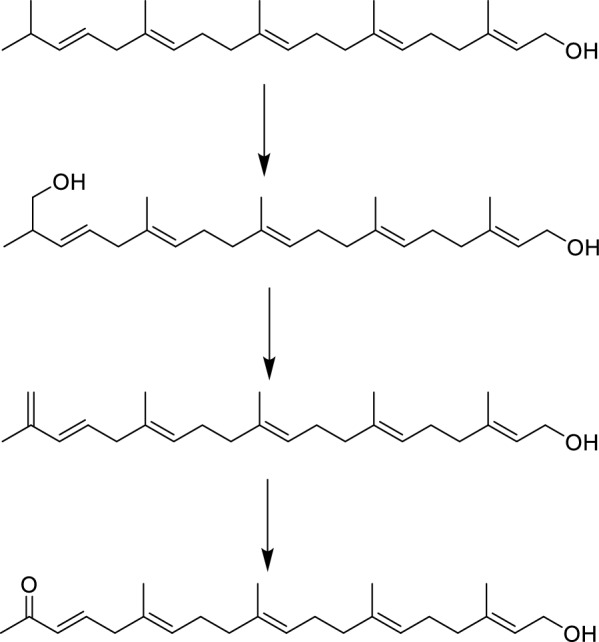
The proposed biogenetic pathway of nicotiazanarpenoid A (**1**)

The detailed analysis of the ^1^H–^1^H COSY and HMQC correlations revealed five spin systems (C-1–C-2, C-4–C-5–C-6, C-8–C-9–C-10, C-12–C-13–C-14, and C-16–C-17–C-18) in compound **1** as shown in bold in Fig. 2. The key HMBCs from H_3_-22 to C-14/C-15/C-16, from H_3_-23 to C-10/C-11/C-12, from H_3_-24 to C-6/C-7/C-8, from H_3_-25 to C-2/C-3/C-4, and from H-20 to C-18/C-19, determined the planar structure of **1**. The geometries of C = C bonds were established as (3*E*), (6*E*), (10*E*), (14*E*), and (18*E*) deduced from the ROESY correlations of H-18/H_2_-16, H_3_-22/H_2_-13, H_3_-23/H_2_-12, H_3_-24/H_2_-5, and H_3_-25/H_2_-1. Structurally, nicotiazanorpenoid A should be a linear 21-norsesterterpenoid. Herein, we propose a putative biogenetic pathway for **1** as shown in Fig. 3.

Through the comparison of their experimental findings with existing literature data, 21 known compounds were recognized as 2,6,11,13-tetradecatetraenal-3,7,13-trimethyl-10-(1-methylethyl) (**2**) [12], scopoletin (**3**) [13], blumenol C (**4**) [14], (6*R*,9*S*)-3-oxo-α-ionol (**5**) [15], isoferulic acid (**6**) [16], 2-phenylethanol (**7**) [17], ferruginol (**8**) [18], 6,7-dehydroferruginol (**9**) [19], phytoprostanes B_1_ type II (**10/11**), phytoprostanes B_1_ type I (**12/13**) [20], Methyl (7*Z*,10*Z*,13*Z*)-7,10,13-hexadecatrienoate (**14**) [21], linolic acid (**15**) [21], 5-(methoxymethyl)-1*H*-pyrrole-2-carboxaldehyde (**16**) [22], methyl 5-hydroxypicolinate (**17**) [23], paraben-acid (**18**) [24], 4-hydroxy-*β*,3-dimethoxybenzeneethanol (**19**), homovanillic alcohol (**20**) [25], *β*-hydroxypropiovanillone (**21**) [26], and 5,6-dihydropyridin-2(1*H*)-one (**22**) [27].

### Bioactivity of compounds from the *N. tabacum*

#### MAO-B inhibitory activity

Although MAO-B inhibitors, such as safinamide, rasagiline, and selegiline [28], have not been demonstrated to alter PD course [29], MAO-B inhibitors may contribute to reduce PD risk. Therefore, 22 compounds were first selected and tested for their MAO-B inhibitory activity in this work. The preliminary screening results were summarized in Table 1, in which five compounds (**3**, **6**, **10/11**, **12/13** and **14**) exhibited inhibition rates above 30% at a concentration of 50 μM, with compounds **3** and **10/11** showing the most substantial effect of around 60%. Further, these compounds were tested for their IC_50_ values against MAO-B through dose–response relationships (Table 1). Compared with the positive control (0.15 ± 0.01 μM), IC_50_ values of compounds **3** and **10/11** were 40.17 ± 1.21 and 37.70 ± 1.57 μM, respectively, and the others ranged from 141.92 to 201.10 μM. This study represents the initial report on the MAO-B inhibitory activity of compounds **10**–**13** derived from *N. tabacum*, which provides a preliminary explanation of the chemical basis of the anti-PD effects of the *N. tabacum.*

**Table 1:** MAO-B inhibitory activities of compounds **1**–**22** and positive control

Compound	Inhibition rate ± SD (%) (50 μM)	IC_50_ (μM) ± SD
**1**	15.45 ± 8.16	–
**2**	11.04 ± 3.99	–
**3**	59.36 ± 4.27	40.17 ± 1.21
**4**	16.49 ± 7.04	–
**5**	24.04 ± 8.05	–
**6**	35.65 ± 5.56	141.92 ± 9.90
**7**	14.45 ± 4.04	–
**8**	17.48 ± 1.50	–
**9**	7.04 ± 4.08	–
**10/11**	68.51 ± 3.26	37.70 ± 1.57
**12/13**	35.89 ± 5.24	173.50 ± 2.57
**14**	31.10 ± 2.54	201.10 ± 7.03
**15**	17.99 ± 4.32	–
**16**	22.21 ± 5.24	
**17**	17.07 ± 5.06	
**18**	8.29 ± 3.90	
**19**	3.71 ± 4.89	
**20**	2.98 ± 2.80	
**21**	11.20 ± 1.81	
**22**	1.05 ± 3.46	
Safinamide	96.87 ± 0.08	0.15 ± 0.01

#### Enzyme kinetic studies

**Fig. 4:**
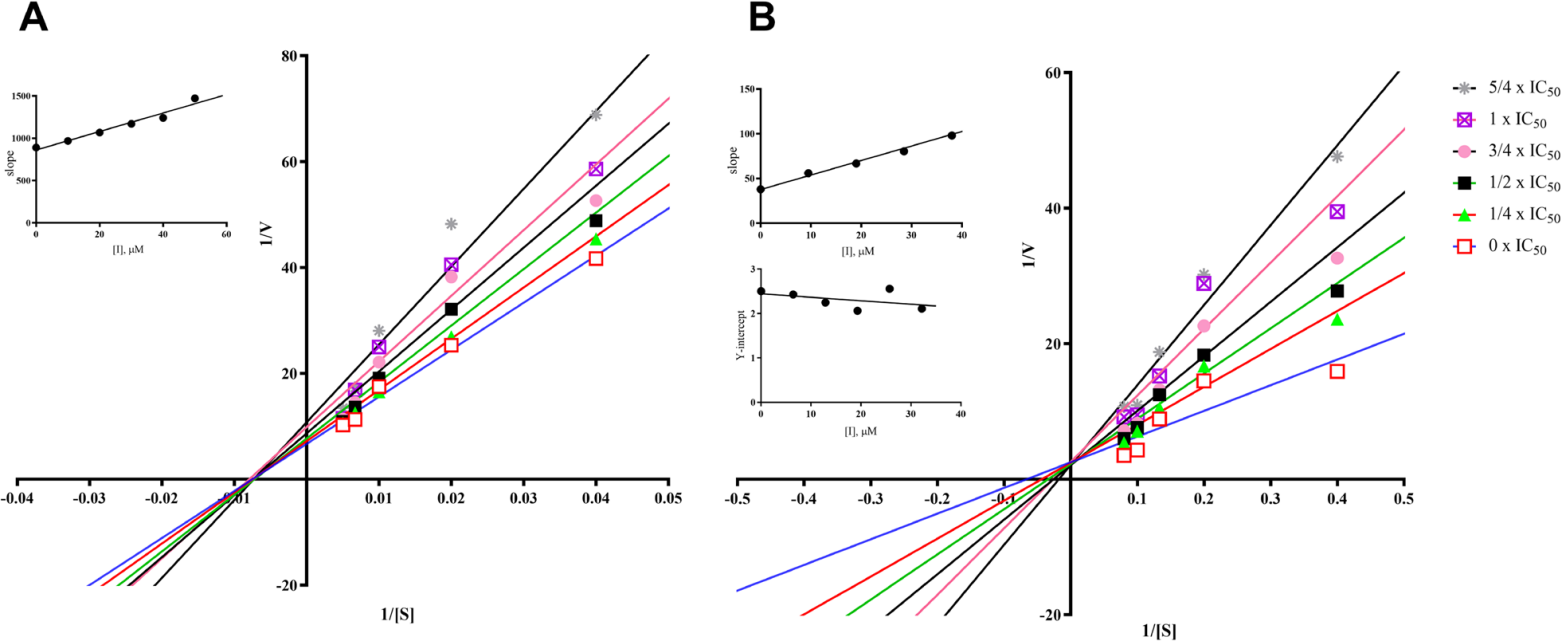
Lineweaver–Burk plots of compounds **3** (**A**) and **10/11** (**B**) against MAO B. (Insets) Replots of the slope and Y-intercept of the Lineweaver–Burk plots versus the inhibitor concentration

Lineweaver–Burk plots were constructed to explore the inhibition mechanism of compounds **3** and **10/11** against MAO-B, as illustrated in Fig. 4. In Fig. 4a, the Lineweaver–Burk plots presented six straight lines intersecting at the X-axis. This phenomenon implies that compound **3** acts as a non-competitive inhibitor of MAO-B. Its *Ki* value, signifying the equilibrium dissociation constant for inhibitor-enzyme binding, was measured at 78.75 μM.

Shifting to Fig. 4b, the Lineweaver–Burk plots for compounds **10**/**11** showed six straight lines intersecting at the Y-axis. This finding indicates a competitive inhibition mechanism against MAO-B, meaning compounds **10**/**11** compete with substrates for the enzyme's active sites. The Ki value (equilibrium constant for inhibitor-enzyme binding) for compounds **10**/**11** was 23.16 μM, while the Kis value (equilibrium constant for inhibitor-substrate-enzyme complex binding) was 310.60 μM. These values suggest that compounds **10**/**11** have a greater affinity for the free enzyme than for the substrate-enzyme complex.

#### Docking studies

**Fig. 5:**
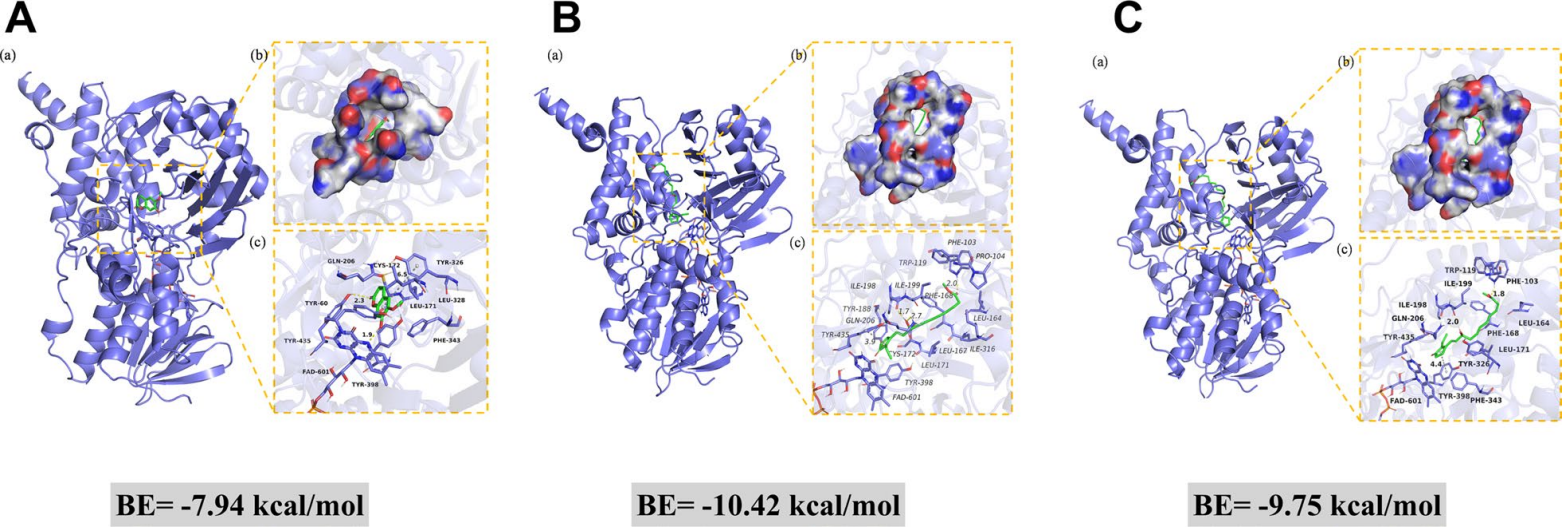
Molecular docking of compounds **3** (**A**), **10** (**B**), and **11** (**C**) with MAO-B. **a** The 3D structure of the complex. **b** The electrostatic surface of the protein. **c** The 3D detail binding mode of the complex. Yellow and gray dash represents hydrogen bond distance or π-stacking

The computational binding modes in 2D and 3D between compounds (**3**, **10**, and **11**) and MAO-B are depicted in Fig. 5. As shown in Fig. 5a, compound **3** forms strong hydrogen bonds with the amino acid residues of Tyr-60 and cofactor FAD-601, and Tyr-326 interacts with the benzene ring of compound **3** through π-π interaction. In Fig. 5b, c, compound **10** exhibits strong hydrogen-bonding with the amino acid residues Trp-119, Ile-198, and Gln-206. The measured hydrogen-bond distances are 2.0 Å, 1.7 Å, and 2.7 Å, respectively, all of which are shorter than the typical 3.5 Å for a hydrogen bond. Besides, compound **10** engages in hydrophobic interactions with several amino acid residues, such as Leu-164, Phe-168, Leu-171, Tyr-326, and Phe-343. Compound **11** interacts with the amino acid residues of Tyr-119 and Ile-199 by hydrogen bonds with the hydrogen bond distances of 2.0 Å and 1.8 Å, respectively. Moreover, compound **11** can form hydrophobic interactions with multiple amino acid residues (ie. Leu-164, Leu-167, Phe-168, Leu-171, Tyr-188, and Ile-316). Both compounds **10** and **11** form π-π conjugation with Tyr-435. The lowest binding energies between **3**, **10**, and **11** to MAO-B were calculated to be − 7.94, − 10.42, and − 9.75 kcal/mol, respectively, which were consistent with their good MAO-B inhibitory activity. The binding energy of **10** is slightly lower than compound **11,** revealing the binding effect between compound **10** and MAO-B is more stable, which may be attributed to compound **10** forming more hydrogen bonds with MAO-B, and it is well-established that hydrogen bonds can enhance binding specificity and reduce binding energy [30, 31].

#### Molecular simulation studies

**Fig. 6:**
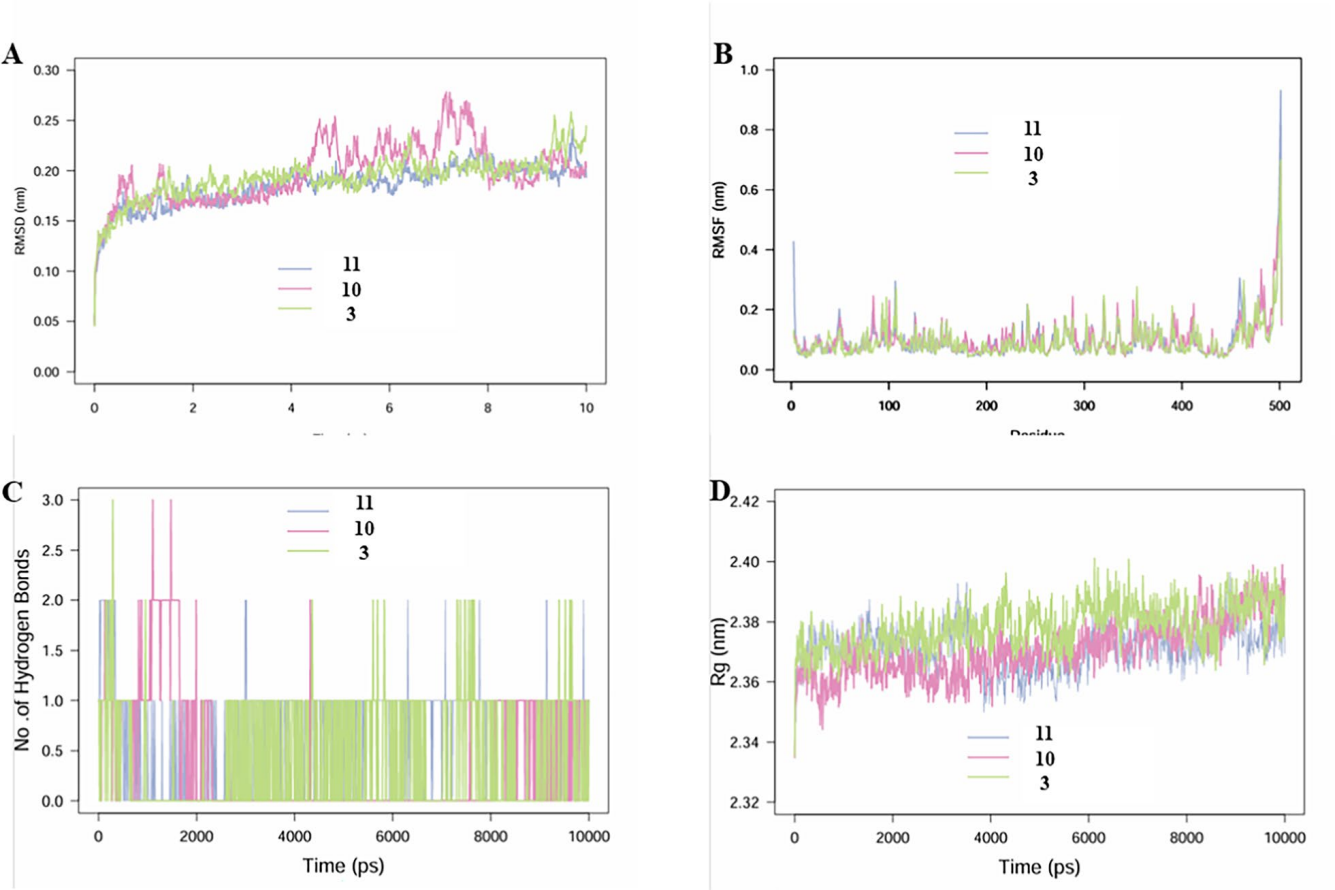
MD simulation analysis of 100 ns trajectories of **A** RMSD of MAO-B bound to ligands CAP. WA. **B** RMSF of MAO-B bound to ligands compounds **3**, **10**, and **11**. **C** Formation of hydrogen bonds in of MAO-B bound to ligand compounds **3**, **10**, and **11**. **D** Rg of MAO-B bound to ligand compounds **3**, **10**, and **11**

Molecular dynamics (MD) simulations were primarily conducted to investigate the complexes formed between the MAO-B protein and compounds **3**, **10**, and **11** (Fig. 6). Root mean square fluctuation (RMSF) analysis revealed that the fluctuations in binding between the three compounds and MAO-B were relatively minor, with the catalytic site remaining stable. Root mean square deviation (RMSD) analysis indicated that the complexes of MAO-B with compounds **3** and **11** exhibited more stable RMSD values, suggesting superior binding efficacy. Although hydrogen bond analysis showed that compound **3** occasionally forms three hydrogen bonds during certain intervals (compared to two for compound **11**), the cumulative count of hydrogen bonds for compound **11** (738) significantly exceeded that of compound **3** (411). This comprehensive evidence demonstrates that compound **11** exhibits the most stable and effective binding among the three compounds.

#### Neuroprotective effects of compounds in 6-OHDA-induced PC12 cells

**Fig. 7:**
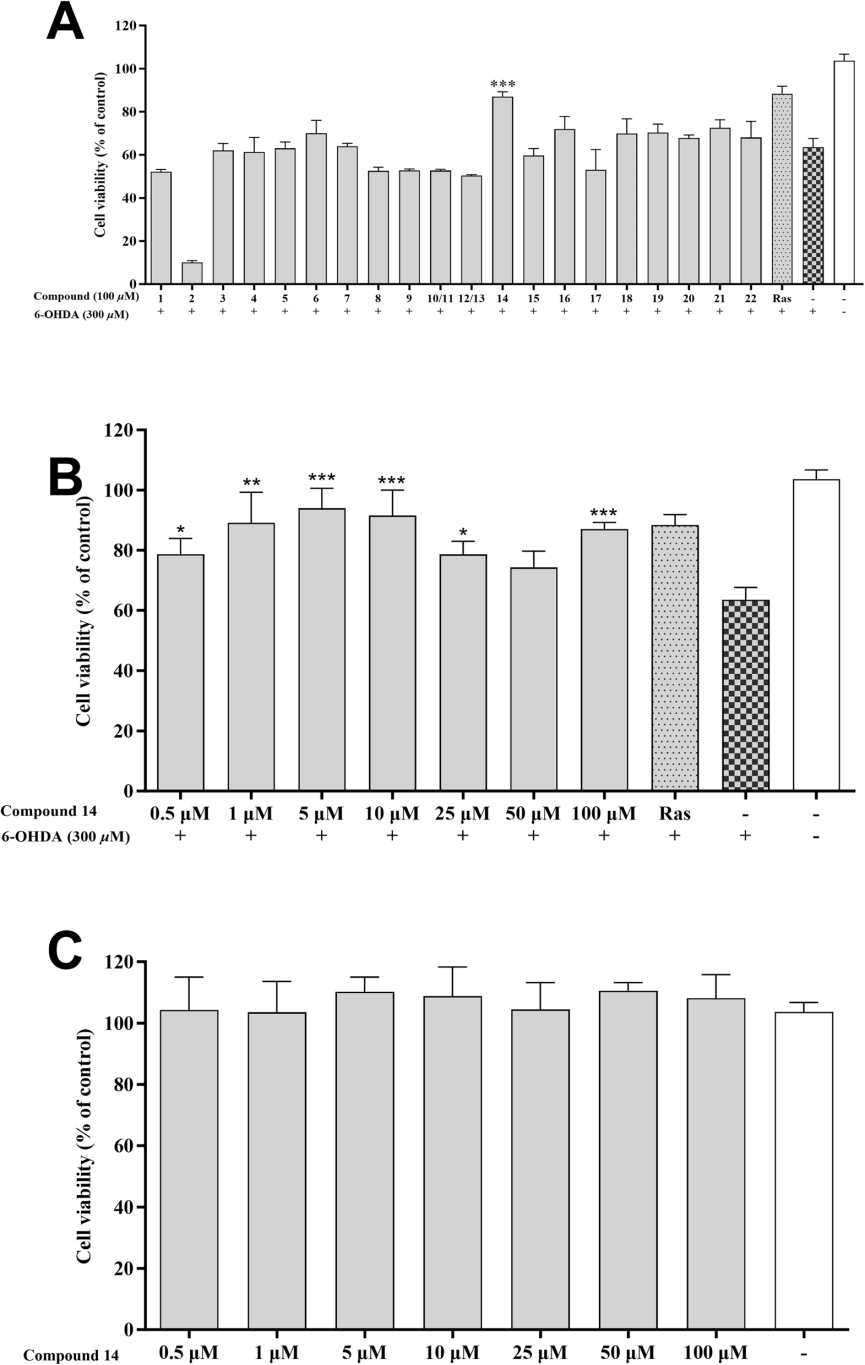
Neuroprotective effects of the isolated compounds against 6-OHDA-induced injury in PC12 cells. **A** Neuroprotective effect of the compounds **1–22** at 100 μM. **B** Neuroprotective effect of compound **14** at various concentrations (0.5, 1, 5, 10, 25, 50, and 100 μM). **C** Cytotoxic effect of compound **14** (0.5, 1, 5, 10, 25, 50, and 100 μM) on PC12 cell. Data were expressed as the means ± SD of three independent experiments. *p < 0.05, **p < 0.01, and ***p < 0.001 compared with model cells in (**A**, **B**) and compared with control cells in (**C**). Rasagiline (Ras) was used as a positive control

The neuroprotective activities of compounds **1**–**22** were further assessed in 6-OHDA-induced PC12 cells. The 6-OHDA is a neurotoxin capable of inducing the death of dopaminergic neurons. It has been extensively utilized to explore the mechanisms underlying PD [32]. Rasagiline has demonstrated effectiveness in protecting against various dopaminergic toxins such as 6-OHDA, MPP^+^, and *β*-amyloid. Therefore, it is often utilized as a positive control in this cellular model [33, 34]. In the preliminary screen (Fig. 7a), compound **14** at 100 μM exhibited an obvious protective effect with cell viability of 87.08%, compared to 63.58% of the model group. Additionally, various concentrations of compound **14** (0.5–100 μM) were tested. As shown in Fig. 7b, **14** enhanced cell viability across all tested concentrations, except 50 μM, and the best concentration was 5 μM (93.94%) in which the cell viability was even higher than that of the rasagiline (88.36%). Furthermore, in the cytotoxicity experiment (Fig. 7c), compound **14** demonstrated extremely low cytotoxicity at different concentrations (0.5–100 μM).

#### Antioxidative activity

The antioxidative activity of the isolated compounds was gauged through DPPH and ABTS assays, with ascorbic acid serving as the positive control. Table 2 shows that among all the isolated compounds, only compound **6** demonstrated DPPH radical scavenging ability, achieving an IC_50_ value of 151.65 ± 1.75 μM. In the ABTS assay, compounds **3**, **6**, **19**, **20**, and **21** exhibited robust radical scavenging capabilities. Their IC_50_ values were 27.74 ± 1.20 μM, 18.13 ± 2.49 μM, 84.06 ± 0.76 μM, 60.51 ± 1.18 μM, and 348.3 ± 17.53 μM respectively. For comparison, ascorbic acid had an IC_50_ of 14.46 ± 0.03 μM in this assay.

**Table 2:** Antioxidant activities of compounds and positive control

Compounds	DPPH		ABTS^+^	
	RSA ± SD (50 μM)	IC_50_ (μM) ± SD	RSA ± SD (50 μM)	IC_50_ (μM) ± SD
**3**	–	–	68.91 ± 1.75	27.74 ± 1.20
**6**	36.00 ± 1.01	151.65 ± 1.75	92.90 ± 0.45	18.13 ± 2.49
**19**	–	–	59.17 ± 3.59	84.06 ± 0.76
**20**	–	–	57.38 ± 1.01	60.51 ± 1.18
**21**	–	–	36.36 ± 1.01	348.3 ± 17.53
Vitamin C	99.97 ± 0.05	9.12 ± 0.25	99.80 ± 0.09	14.46 ± 0.03

These findings indicate that the antioxidative properties of these compounds might be the source of their neuroprotective effects, which warrants further exploration.

## Discussion

This study reveals the low-polarity chemical profile of nicotine-ultra-low *N. tabacum*, and its multi-target neuroprotective models by integrating phytochemical and pharmacological approaches. Among the 22 compounds identified, Nictiazanorpenoid A (**1**) represents the first 21-norsesterterpenoid found in the Solanaceae family, while eight ones were discovered in *N. tabacum* for the first time. Additionally, five compounds show inhibitory activity against MAO-B, two possess anti-oxidative properties in the ABTS radical scavenging assay, and one performs good neuroprotective activity to PC-12 cells effectively against 6-OHDA-induced cytotoxicity. Notably, we demonstrate for the first time that plant prostaglandins, which were previously recognized solely for their defensive functions [35], exhibit moderate MAO-B inhibition activity. Their activities suggest that these compounds could serve as valuable chemical probes for structure–activity relationship studies. Moreover, their natural origin makes them particularly interesting for investigating plant-derived neuromodulators. The most abundant scopoletin (**3**), exhibited selective (and reversible) inhibition of human (*Ki* = 20.7 μM) and murine (*Ki *= 22 μM) MAO-B, demonstrating approximately 3.5-fold selectivity for MAO-B over MAO-A. In *vivo*, intraperitoneal administration of scopoletin (**3**) (80 mg/kg) significantly elevated dopamine levels while reducing the striatal metabolite 3,4-dihydroxyphenylacetic acid (DOPAC) in treated subjects [36]. Additionally, compound **14** exhibited superior neuroprotective effects, potentially mediated by synergistic activation of the Nrf2/ARE signalling pathway and inhibition of oxidative stress. It was previously reported that some similar compounds (such as DHA) might have similar activities. [37]. Importantly, the anti-PD compounds identified in this study provide a new platform for producing plant-derived multi-target neuroprotectants, potentially solving the single-target MAO-B inhibitory tolerance issue. The synergistic compound system within *N. tabacum* extracts may achieve sustained therapeutic efficacy.

## Conclusion

In this study, a systematic separation and identification of the low-polarity chemical components of ultra-low-nicotine *N. tabacum* was conducted. 22 compounds were isolated and identified, including the first discovery of a new linear 21-norsesterterpenoid. Additionally, through various activity evaluation models, five compounds with MAO-B inhibitory activity, one compound with neuroprotective activity, and two compounds with ABTS radical scavenging activity were discovered in the *N. tabacum* These findings suggest that *N. tabacum* harbours multiple neuroprotective activities, including MAO-B inhibition and antioxidative effects, which may contribute to reducing the risk of Parkinson’s disease and potentially other neurodegenerative disorders.

## Materials and methods

### Plant materials

The ultra-low nicotine *N. tabacum* was provided by Dr. Xue-Yi Sui [11].

### General experimental procedures

Nuclear magnetic resonance (NMR) spectra, including ^1^H, ^13^C, DEPT, ^1^H-^1^H COSY, HSQC, HMBC, and ROESY, were recorded using a Bruker Avance III 500 spectrometer (Bruker, Zurich, Switzerland), employing tetramethylsilane (TMS) as the internal standard. Infrared (IR) measurements were obtained with a Bruker Tensor-27 spectrometer (Bruker, Germany). High-resolution electrospray ionization mass spectrometry (HRESIMS) analyses were conducted on an Agilent Q-TOF mass spectrometer (Agilent, Redwood City, CA, USA). Ultraviolet (UV) data was collected using a thermal multimode microplate reader (Waltham, MA, USA). Column chromatography was carried out using MCI gel (CHP-20P, Mitsubishi Chemical Industries Co., Ltd., Japan), silica gel (200-300 mesh, Qingdao Marine Chemical Plant, Qingdao, China), and reversed-phase C18 silica gel (YMC Group). Analytical high-performance liquid chromatography (HPLC) was performed on Waters 2695 (Waters Associates, Massachusetts, USA) and Shimadzu LC-20 (Shimadzu, Japan) HPLC systems. The MAO-B inhibition assay was conducted by a Thermo Scientific Varioskan Flash (Waltham, MA, USA). The instruments, reagents, and general experimental procedures used in this study were the same as those detailed in our previous reports [38, 39].

### Extraction and isolation

The dried leaves of the *N. tabacum* were ground into a powder weighing 28 kg. This powder underwent extraction three times with 50 L of methanol at ambient temperature. After extraction, the filtrates from each round were combined and evaporated under reduced pressure, resulting in 10 kg of extract. The extract was processed using silica gel column chromatography (CC) with CH₂Cl₂/MeOH eluent (1:0, 500:1, 200:1, 50:1, 20:1, 5:1, 0:1, v/v), producing seven distinct fractions (Fr. 1–Fr. 7).

Fr. 3, with a mass of 146.4 g, was further partitioned using MCI gel CC employing a MeOH/H₂O eluent (from 3:10 to 1:0, v/v), producing eight subfractions (Fr. 3.1–Fr. 3.8). Sub-fraction Fr. 3.6 (5.6 g) underwent chromatographic separation on Sephadex LH-20 with a mobile phase CH₂Cl₂/MeOH (1:1) and then purified by semi-preparative HPLC using MeCN/H₂O (6:10, v/v, 2 mL/min). This procedure resulted in the isolation of compounds **8** (8.6 mg,* t*_R_ 23 min), **9** (4.3 mg, t_R_ 28 min), and **22** (12.3 mg,* t*_R_ 32 min).

Compound **3** was directly crystallized from Fr. 4. The supernatant of Fr. 4, weighing 455.7 g, was further fractionated using MCI gel CC with MeOH/H₂O (from 3:10 to 1:0, v/v), giving eight fractions (Fr. 4.1–Fr. 4.8). Sub-fraction Fr. 4.4 was partitioned using Sephadex LH-20 with MeOH and then further purified by semi-preparative HPLC with a solvent system of n-Hexane/isopropanol (1:10, v/v, 2 mL/min), yielding compounds **4** (8.4 mg,* t*_R_ 18 min) and **5** (5.5 mg,* t*_R_ 19 min). Subfraction Fr. 4.6 was chromatographed on silica gel CC with CH₂Cl₂/MeOH (from 200:1 to 0:1, v/v), resulting in eight subfractions (Fr. 4.6.1–Fr. 4.6.8). Fr. 4.6.4 was fractionated on Sephadex LH-20 (CH₂Cl₂/MeOH, 1:1, v/v) and then purified by semi-preparative HPLC with MeCN/H₂O (30:70, v/v, 2 mL/min), yielding compounds **1** (5.6 mg,* t*_R_ 15 min), **14** (6.5 mg,* t*_R_ 21 min), and **15** (11.1 mg,* t*_R_ 8 min).

Fr. 5, weighing 137.0 g, was further fractionated into five subfractions (Fr. 5.1–Fr. 5.5) using MCI gel CC with MeOH/H₂O (from 6:10 to 1:0, v/v). The subfraction Fr. 5.2, weighing 12.6 g, was first purified by silica gel CC with CH₂Cl₂/MeOH (20:1, v/v) and then by semi-preparative HPLC with MeCN/H₂O (6:10, v/v, 2 mL/min). This purification process led to the isolation of compounds **2** (23.0 mg,* t*_R_ 45 min), **6** (32.6 mg,* t*_R_ 26 min), **7** (30.6 mg,* t*_R_ 18 min), **10**/**11** (2.6 mg,* t*_R_ 22 min), **12**/**13** (2.5 mg,* t*_R_ 24 min), **16** (8.9 mg,* t*_R_ 23 min), **17** (5.2 mg,* t*_R_ 25 min), **18** (3.3 mg,* t*_R_ 20 min), **19** (12.6 mg,* t*_R_ 28 min), **20** (15.6 mg,* t*_R_ 32 min), and **21** (2.0 mg,* t*_R_ 35 min).

#### Nicotiazanorpenoid A (1)

Colorless oil; IR (KBr) *ν*_max_ 3446, 3437, 2959, 2921, 2853, 1637, 1543, 1456, 1435, 1420, 1384, 1316, 1261, 1164, 1095, 1047, 1030, 877, 860, 800, 675, 661, 619, and 421 cm^− 1^; UV (MeOH) *λ*_max_ (log *ε*) 196 (0.50) nm; ^1^H and ^13^C NMR (CDCl_3_) spectra data see Table 3; HRESIMS: *m/z* 381.2768 [M + Na]^+^ (calcd for C_24_H_38_O_2_Na, 381.2764).

**Table 3:** ^1^H (500 MHz) and ^13^C (125 MHz) NMR data of compound **1** in CDCl_3_

1					
Pos.	*δ*_C,_ type	*δ*_H_ mult. (*J* in Hz)	Pos.	*δ*_C,_ type	*δ*_H_ mult. (*J* in Hz)
1	59.6, CH_2_	4.16, d (6.5)	13	26.4, CH_2_	1.96–2.12, m
2	123.5, CH	5.42, t (7.0)	14	127.6, CH	5.19, t (7.0)
3	139.9, C	–	15	131.4, C	–
4	39.9, CH_2_	1.96–2.12, m	16	42.7, CH_2_	2.86, d (7.0)
5	26.9, CH_2_	1.96–2.12, m	17	146.7, CH	6.76, dt (16.0, 7.0)
6	124.0, CH	5.11, t (6.5)	18	132.2, CH	6.06, d (16.0)
7	135.5, C	–	19	198.9, C	–
8	39.7, CH_2_	1.96–2.12, m	20	27.0, CH_3_	2.25, s
9	26.8, CH_2_	1.96–2.12, m	22	16.4, CH_3_	1.61, s
10	124.7, CH	5.11, t (6.5)	23	16.2, CH_3_	1.60, s
11	134.8, C	–	24	16.2, CH_3_	1.60, s
12	39.6, CH_2_	1.96–2.12, m	25	16.5, CH_3_	1.68, s

### MAO-B assays

The MAO-B inhibition assay was performed on 96-well microtiter plates according to the procedure detailed in our prior report [40]. In preparing the microplates, 50 μL of MAO-B (2.5 U/mL) was combined with 100 μL of the test compounds at different concentrations. This mixture was incubated at 37 °C for 10 min. DMSO was the negative control, while safinamide was the positive control. Next, 50 μL of kynuramine (0.2 mM) sourced from Macklin in Shanghai, China, was added to the mixture. The resulting mixture was incubated at 37 °C for an additional 30 min. The reaction was halted by adding 80 μL of 2 N NaOH. Ultimately, the enzyme activity was measured using a microplate reader, with the excitation and emission wavelengths set at 310 nm and 400 nm, respectively.

### Kinetic studies of MAO-B inhibition

The kinetic study of MAO-B inhibition was the Lineweaver–Burk plot, which was performed using the Lineweaver–Burk curve method. Compounds **3** and **10/11** with six different concentrations (0, 1/4 × IC_50_, 1/2 × IC_50_, 3/4 × IC_50_, 1 × IC_50_, and 5/4 × IC_50_) were added into the assay solution with a series of increasing concentrations of kynuramine. Kinetic characterization of their MAO-B inhibition was recorded 20 min after initiation. Constants *Kis* and *Ki* were calculated using the Lineweaver–Burk plots.

### Docking studies

Docking studies were performed to explore the interactions between compounds **3** and **10**/**11** with MAO-B [41]. Among them, compounds **10** and **11** are a pair of enantiomers. Compounds **3**, **10** and **11** were constructed in ChemDraw 4.5, and their three-dimensional (3D) structures were optimized by Chem3D 4.5 for molecular energy minimization using the MM2 force field. Structural data for MAO-B (PDB ID: 6YT2) was obtained from the Protein Data Bank (https://www.rcsb.org/structure). Molecular docking was conducted using the Glide functionalities within Schrödinger Maestro software.

### Molecular simulation studies

Molecular dynamics (MD) simulations were performed using Desmond 2020.1 (Schrödinger) to investigate the interactions of compounds 3, 10, and 11 with MAO-B [42]. The OPLS-2005 force field and TIP3P explicit solvent model were applied within a periodic boundary solvation box (10 × 10 × 10 Å). Protein–ligand complexes underwent structural optimization and energy minimization via the Protein Preparation Wizard, followed by system assembly using the System Builder tool. Equilibration involved a 10 ns NVT ensemble phase for conformational stabilization and a subsequent 12 ns NPT ensemble phase for pressure–temperature equilibration, with temperature (300 K) and pressure (1 atm) regulated by the Nose–Hoover thermostat. Long-range electrostatic interactions were computed using the Particle Mesh Ewald (PME) method (9 Å cutoff), while pressure control employed the Martyna-Tuckerman-Klein scheme with a 2-fs timestep. A 100 ns production simulation was conducted, with system stability assessed through root mean square deviation (RMSD), radius of gyration (Rg), residue-specific root mean square fluctuation (RMSF), and hydrogen bond occupancy analysis, enabling comprehensive evaluation of ligand binding dynamics and structural integrity.

### Cell viability assays

Two-dimensional (2D) cell culture models were used for performing cell viability assays [38]. Approximately 10,000 PC12 cells (Fenghui, Changsha, China) were seeded into each well of a 96-well plate. The cells underwent pre-treatment with the test compounds for 2 h at 37 °C. After this pre-treatment step, the cells were subjected to 300 μM 6-OHDA (Aladdin, Shanghai, China) for 24 h. The plate was incubated at 37 °C for 1 h after adding 10 μL of CCK-8 solution (ProteinTech, Chicago, USA) to each well. Then, each well's optical density (OD) was measured at 450 nm using a multimode microplate reader (Waltham, MA, USA).

### Antioxidative activity

The antioxidative capacity of the obtained compounds was carried out through DPPH radical scavenging assays and ABTS assay (Macklin, Shanghai, China) with some modifications, and Vitamin C was used as the positive control [43–45]. In the DPPH assay, 60 μL of the isolates at varying concentrations were mixed with 100 μL of 100 μM DPPH solution in ethanol in a 96-well microplate. The mixture was shaken for 10 s and then allowed to sit in darkness at 30 °C for 5 min. Subsequently, the absorbance was determined using a microplate reader at a wavelength of 517 nm. The radical-scavenging activity of the compounds was assessed using the formula RSA (%) = [(AB-AA)/AB] × 100%, where AA was the absorbance of the sample and AB was the absorbance of the blank sample. The IC_50_ value was calculated with GraphPad Prism 7.0, and all experiments were conducted in triplicate.

In the ABTS^+^ assay, the ABTS solution was diluted using 95% methanol until it had an absorbance of 0.7 ± 0.02 at 734 nm. Various concentrations of the compounds (60 μL each) were then combined with 150 μL of the diluted ABTS^+^ solution. The reaction was maintained in the dark at 30 °C for 6 min. Afterwards, the absorbance was determined at 734 nm with a microplate reader. The calculation of ABTS radical scavenging activity followed the same method as for DPPH radical scavenging activity. IC_50_ values were determined using GraphPad Prism 7.0, and all tests were conducted in triplicate.

### Chromatographic and mass spectrometric conditions

The dried leaves of the flue-cured Yunyan 87 and *Nic1-2Nic2-2* were freeze-dried and subjected to ultra-fine grinding. 10.0 g aliquot of the *N. tabacum* leaf powder was accurately weighed, and methanol solvent was added at a solid-to-liquid ratio of 1:5 (w/v). Ultrasonic-assisted extraction was performed for 30 min per cycle. After filtration through a membrane filter, the extraction process was repeated three times. The three extracts were combined and concentrated under reduced pressure using a rotary evaporator to obtain the methanolic extract paste.

The chromatographic column was Luna Omega 3 μm Polar C18 100 Å 100 × 2.1 mm. The flow rate was 0.3 ml/min, the column temperature was 25 °C, the injection volume was 1 μL, the mobile phase A was 0.1% formic acid water, and the mobile phase B was acetonitrile. The gradient elution conditions were as follows: 0–15 min (5–95% B), 15–20 min (95% B), 20–25 min (95–5% B), 25–30 min (5% B).

The mass spectrometry conditions were as follows: electrospray ionization source (ESI) positive ion mode, de-custering potential (DP): 80 V; collision energy (CE): 40 ± 10 eV; curtain gas (CUR): 35 psi; ion source gas 1 (GS1): 55 psi; ion source gas 2 (GS2): 55 psi; ion source temperature: 500 °C; ion spray voltage floating (SVF): 5500; primary scan mode: Full MS; scan range: 100–2000 m*/z*; secondary scan mode: Full MS/dd-MS2; scan range: 50–2000 m*/z*.

### Statistical analysis

One-way analysis of variance (ANOVA) was used to evaluate statistical significance, followed by Dunnett's multiple comparisons test, and data analysis was performed using GraphPad Prism 7.0 software. The levels of significance are represented as follows: ^*^p < 0.05, ^**^p < 0.001, and ^***^p < 0.0001.

## Supplementary Information

Additional file 1.

## Data Availability

The data underlying this study are available in the published article and its online supporting information.
