# Supplementary material for: Neuroprotective effects of chemical constituents from Nicotiana tabacum L. in Parkinson’s disease
Source: Nat Prod Bioprospect. 2025 Oct 1;15(1):67. doi: 10.1007/s13659-025-00541-8 (PMC12484522; doi:10.1007/s13659-025-00541-8)
Supplement: Supplementary file 1 — Additional file 1. [file 13659_2025_541_MOESM1_ESM.docx]

**Supplementary material**

**Neuroprotective effects of chemical Constituents from Nicotiana tabacum L. in Parkinson’s disease**

Hao-Jing Zang^a,c,d,‖^, Xiao-Lin Bai ^b,d,‖^, Xue-Yi Sui ^e,‖^, Xiao-Rui Zhai ^b, d^, Yong-Cui Wang^a^, Zhong-Quan Xin^a^, Qiu-Yuan Ying ^c^, Xiao-Jiang Hao ^a,f,g^, Yue-Hu Wang ^h,^*, Xun Liao ^b^ *, Ying-Tong Di ^a^ *

^a^ State Key Laboratory of Phytochemistry and Natural Medicines, Kunming Institute of Botany, Chinese Academy of Sciences, Kunming 650201, China

^b^ Chengdu Institute of Biology, Chinese Academy of Sciences, Chengdu 610041, China

^c^ School of Life Sciences, Yunnan University, Kunming 650091, China

^d^ University of Chinese Academy of Sciences, Beijing 100049, China

^e^ National Tobacco Genetic Engineering Research Center, Yunnan Academy of Tobacco Agricultural Sciences, Kunming 650021, China

^f^ Research Unit of Chemical Biology of Natural Anti-Virus Products, Chinese Academy of Medical Sciences, Beijing, 100730, *China*

^g^ Yunnan Characteristic Plant Extraction Laboratory, Kunming 650201, *China*

^h^ Yunnan Key Laboratory for Wild Plant Resources, Kunming Institute of Botany, Chinese Academy of Sciences, Kunming, 650201, *China*

^‖^ These authors contributed equally to this work.

* Corresponding authors:

Dr. Ying-Tong Di, Email: diyt@mail.kib.ac.cn; Tel: +86-871-65223263

*Dr. Xun Liao*, Email: liaoxun@cib.amc.cn; Tel: +86-28-82890402

*Dr. Yue-Hu Wang*, Email: [wangyuehu@mail.kib.ac.cn](mailto:wangyuehu@mail.kib.ac.cn); Tel: +86-871-65223214

**Contents**

[Figure S1. ^1^H NMR spectra (CDCl_3_, 500 MHz) of nicotiazanarpenoid A (**1**) 3](#_Toc205917708)

[Figure S2. ^13^C and DEPT NMR spectra (CDCl_3_, 125 MHz) of nicotiazanarpenoid A (**1**) 3](#_Toc205917709)

[Figure S3. ^1^H-^1^H COSY spectra (CDCl_3_, 500MHz) of nicotiazanarpenoid A (**1**) 4](#_Toc205917710)

[Figure S4. HSQC spectra (CDCl_3_, 500MHz) of nicotiazanarpenoid A (1) 4](#_Toc205917711)

[Figure S5. HMBC spectra (CDCl_3_, 500MHz) of nicotiazanarpenoid A (1) 5](#_Toc205917712)

[Figure S6. ROESY spectra (CDCl_3_, 500MHz) of nicotiazanarpenoid A (1) 5](#_Toc205917713)

[Figure S7. UV spectra of nicotiazanarpenoid A (1) 6](#_Toc205917714)

[Figure S8. IR spectra of nicotiazanarpenoid A (1) 6](#_Toc205917715)

[Figure S9. ORD spectra of nicotiazanarpenoid A (1) 7](#_Toc205917716)

[Figure S10. HR-ESI-MS of nicotiazanarpenoid A (1) 8](#_Toc205917717)

[Figure S11. ^1^H NMR spectra (CDCl_3_, 500 MHz) of compound 19 9](#_Toc205917718)

[Figure S12. ^13^C NMR spectra (CDCl_3_, 500 MHz) of compound 19 9](#_Toc205917719)

[Figure S13. ^1^H-^1^H COSY spectra (CDCl_3_, 500 MHz) of compound 19 10](#_Toc205917720)

[Figure S14. HSQC spectra (CDCl_3_, 500 MHz) of compound 19 10](#_Toc205917721)

[Figure S15. HMBC spectra (CDCl_3_, 500 MHz) of compound 19 11](#_Toc205917722)

[Figure S16. ROESY spectra (CDCl_3_, 500 MHz) of compound 19 11](#_Toc205917723)

[Table S1. ^1^H (500 MHz) and ^13^C (125 MHz) NMR data of compound 19 in CDCl_3_. 12](#_Toc205917724)

[Figure S17. ORD spectra of 10/11 13](#_Toc205917725)

[Figure S18. ORD spectra of 12/13 13](#_Toc205917726)

[Figure S19. HPLC spectra of 10/11 and 12/13 14](#_Toc205917727)


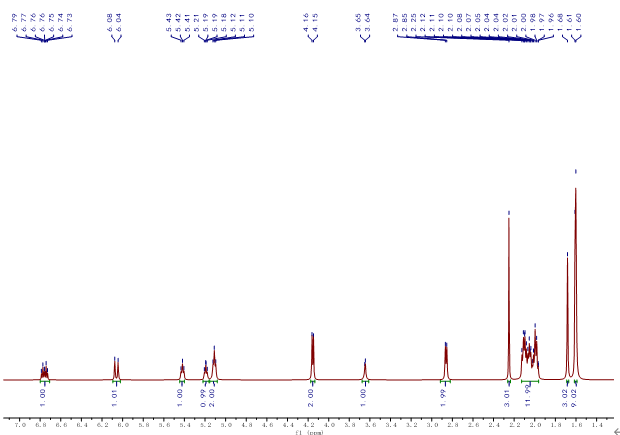


# Figure S1. ^1^H NMR spectra (CDCl_3_, 500 MHz) of nicotiazanarpenoid A (**1**)


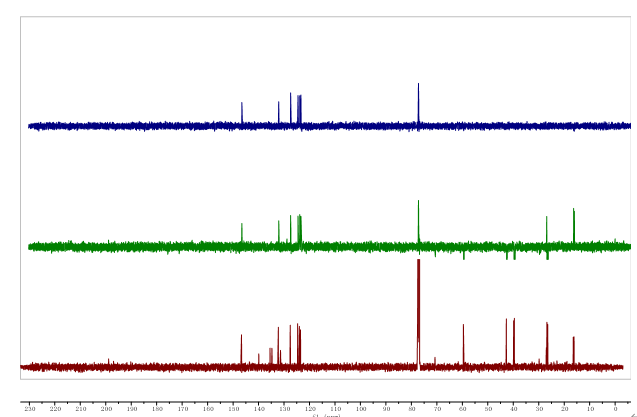


Figure S2. ^13^C and DEPT NMR spectra (CDCl_3_, 125 MHz) of nicotiazanarpenoid A (**1**)


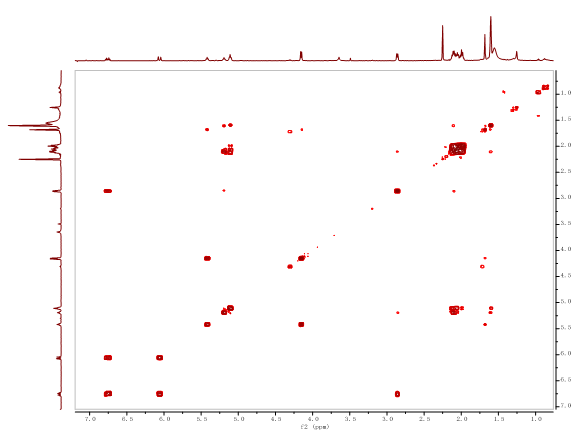


Figure S3. ^1^H-^1^H COSY spectra (CDCl_3_, 500MHz) of nicotiazanarpenoid A (**1**)


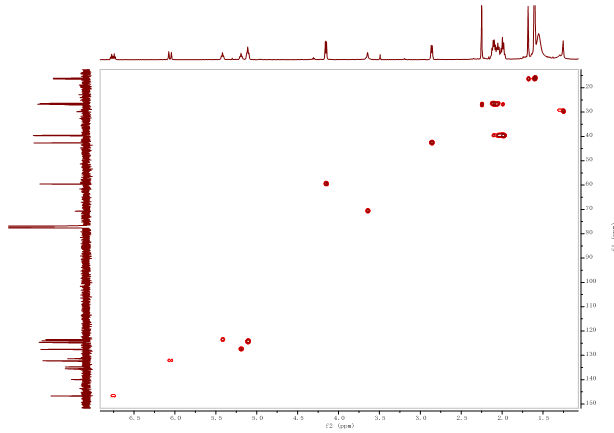


Figure S4. HSQC spectra (CDCl_3_, 500MHz) of nicotiazanarpenoid A (1)


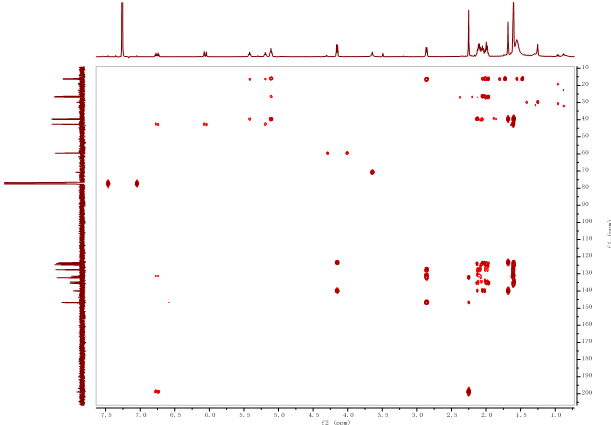


Figure S5. HMBC spectra (CDCl_3_, 500MHz) of nicotiazanarpenoid A (1)


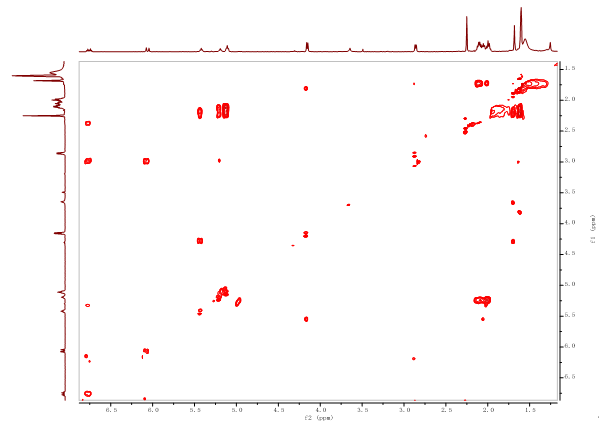


Figure S6. ROESY spectra (CDCl_3_, 500MHz) of nicotiazanarpenoid A (1)


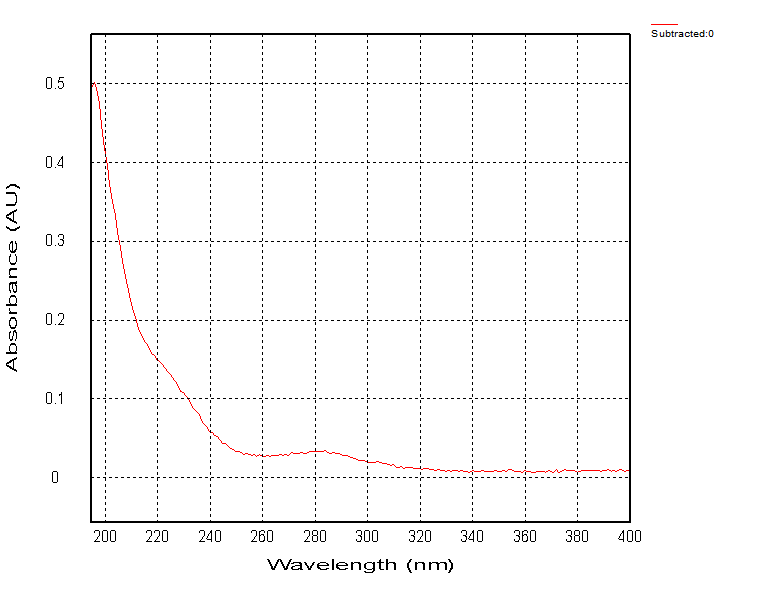


Figure S7. UV spectra of nicotiazanarpenoid A (1)

Figure S8. IR spectra of nicotiazanarpenoid A (1)


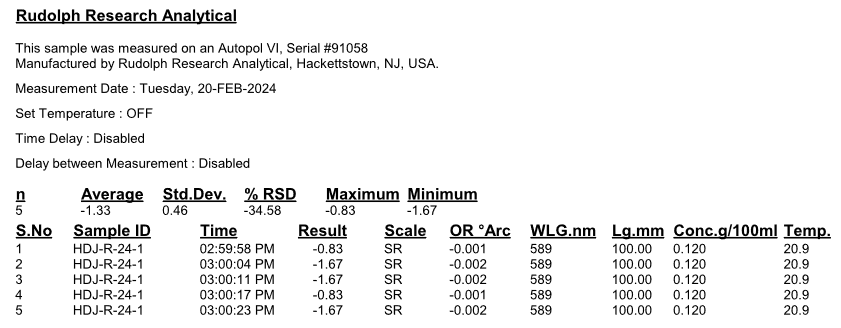


Figure S9. ORD spectra of nicotiazanarpenoid A (1)

**
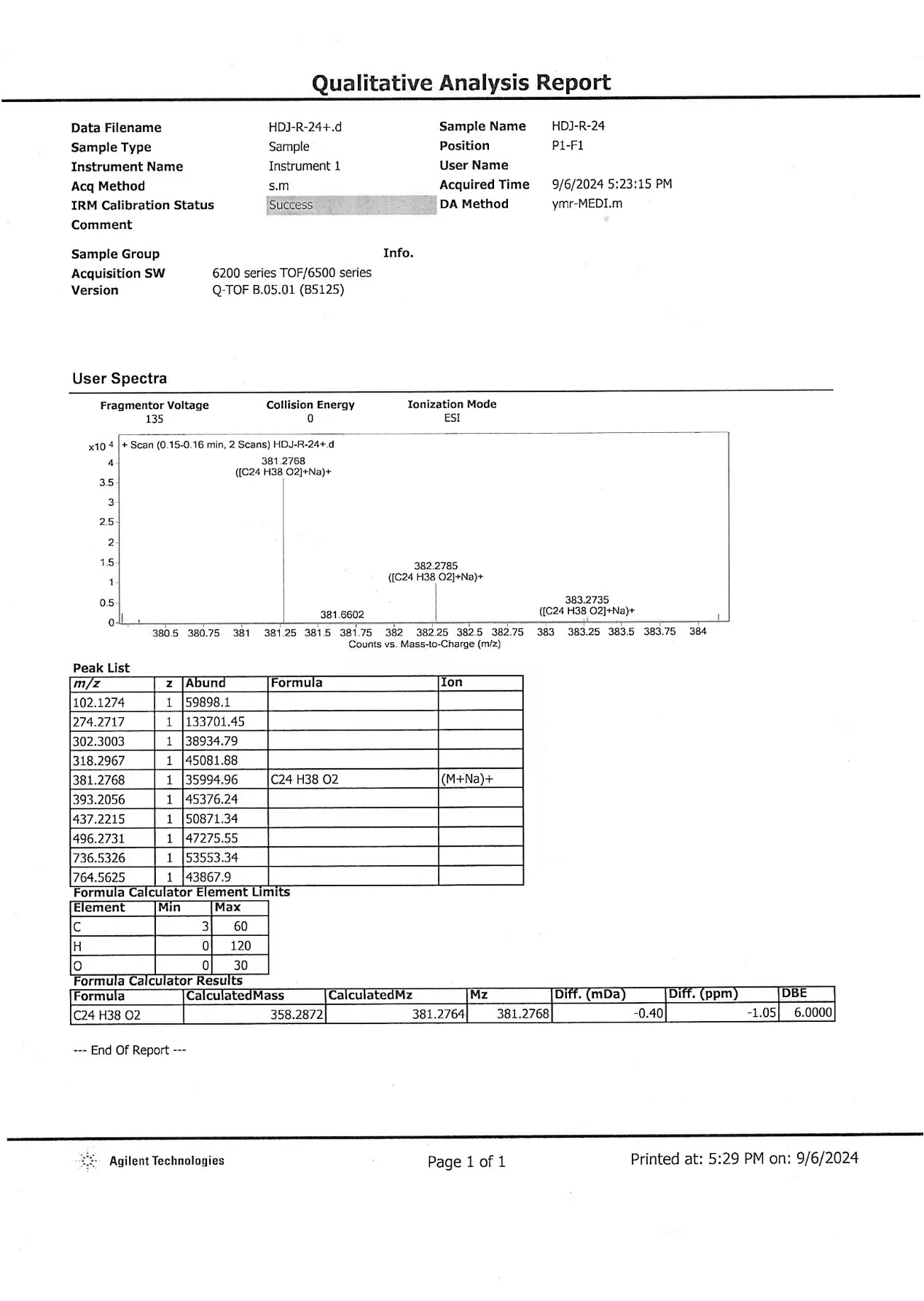
**

Figure S10. HR-ESI-MS of nicotiazanarpenoid A (1)


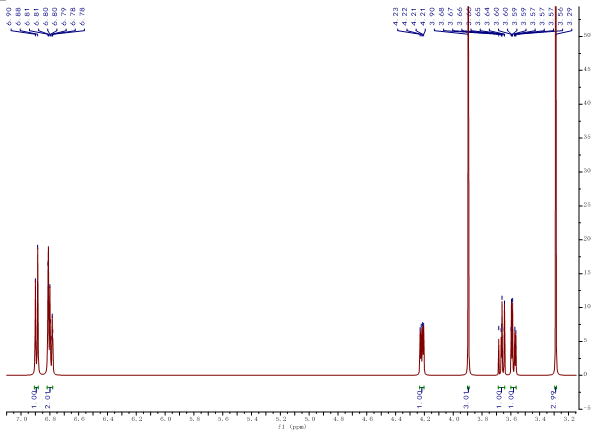


Figure S11. ^1^H NMR spectra (CDCl_3_, 500 MHz) of compound 19


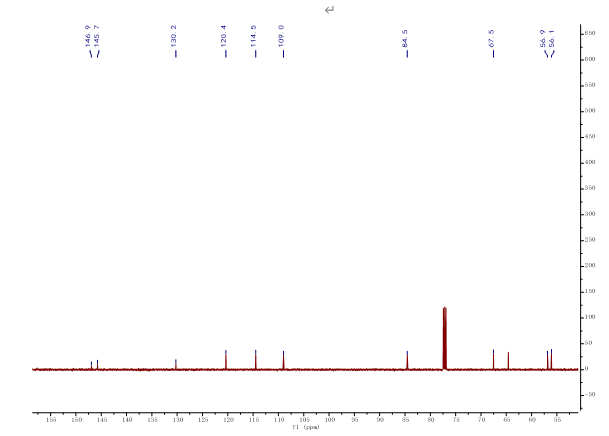


Figure S12. ^13^C NMR spectra (CDCl_3_, 500 MHz) of compound 19


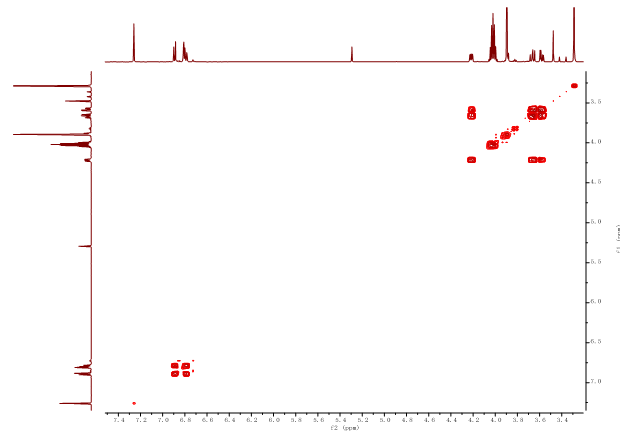


Figure S13. ^1^H-^1^H COSY spectra (CDCl_3_, 500 MHz) of compound 19


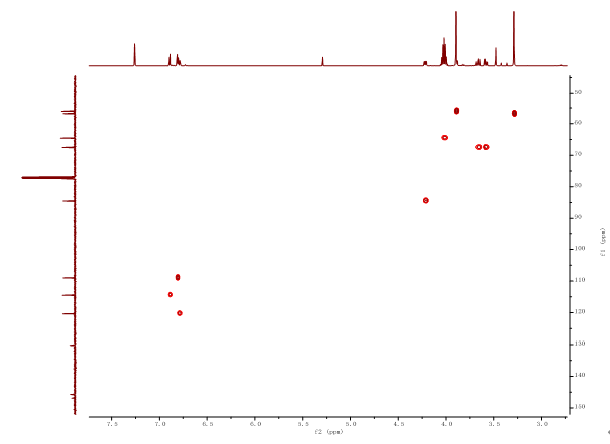


Figure S14. HSQC spectra (CDCl_3_, 500 MHz) of compound 19


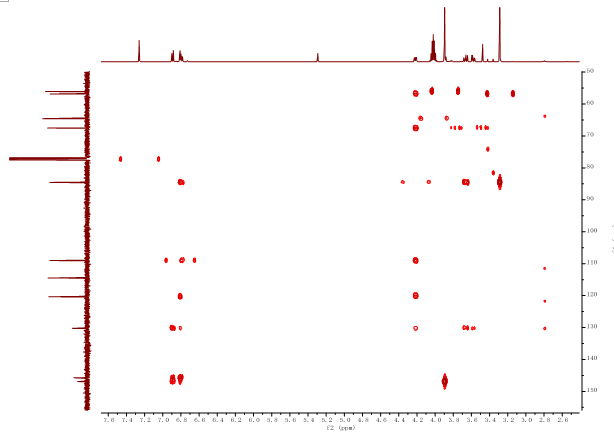


Figure S15. HMBC spectra (CDCl_3_, 500 MHz) of compound 19


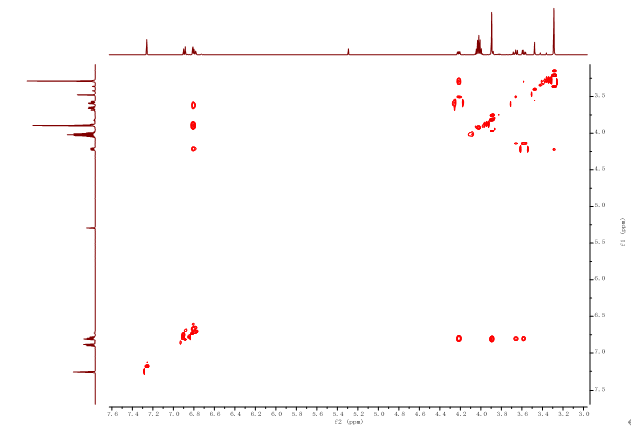


Figure S16. ROESY spectra (CDCl_3_, 500 MHz) of compound 19

Table S1. ^1^H (500 MHz) and ^13^C (125 MHz) NMR data of compound 19 in CDCl_3_.

|  | **19** | |
| --- | --- | --- |
| pos. | *δ*_C,_ type | *δ*_H_ mult. (*J* in Hz) |
| 1 | 145.7, C | - |
| 2 | 146.9, C | - |
| 3 | 114.5, CH | 6.89, d (8.0) |
| 4 | 120.4, CH | 6.78 ̶ 6.81, m |
| 5 | 130.3, C | - |
| 6 | 109.0, CH | 6.78 ̶ 6.81, m |
| 7 | 84.6, CH | 4.22, dd (8.5, 4.0) |
| 8 | 67.5, CH_2_ | 3.68, m, 3.58, m |
| 1-OMe | 56.1, CH_3_ | 3.88, s |
| 7-OMe | 56.9, CH_3_ | 3.27, s |


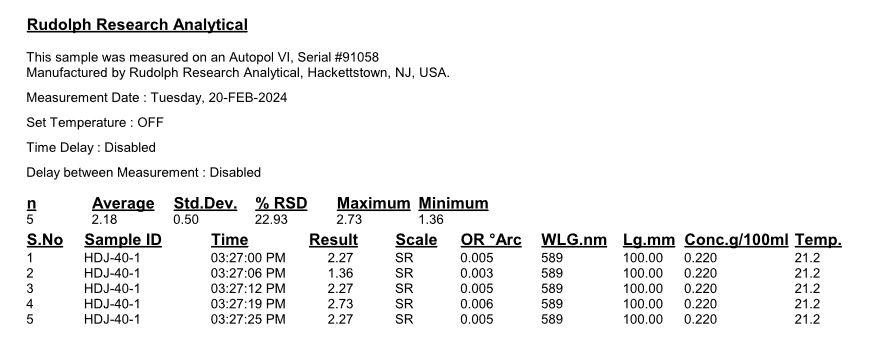


Figure S17. ORD spectra of 10/11


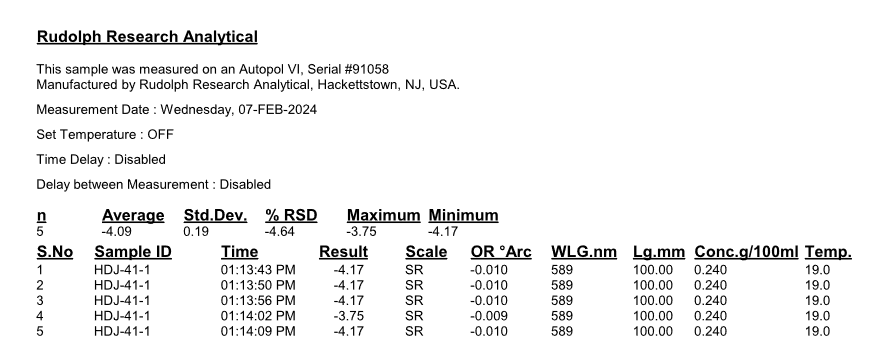


Figure S18. ORD spectra of 12/13

**chromatographic condition**：Chromatographic separation was performed using a CHIRALPAK AD-RH (5 μm 250 × 4.6 mm) maintained at 25 °C. The mobile phase consisted of (A) water and (B) acetonitrile, delivered at a flow rate of 1.0 mL/min with an injection volume of 10 μL. The gradient elution program was as follows: 0–30 min, 60% B.


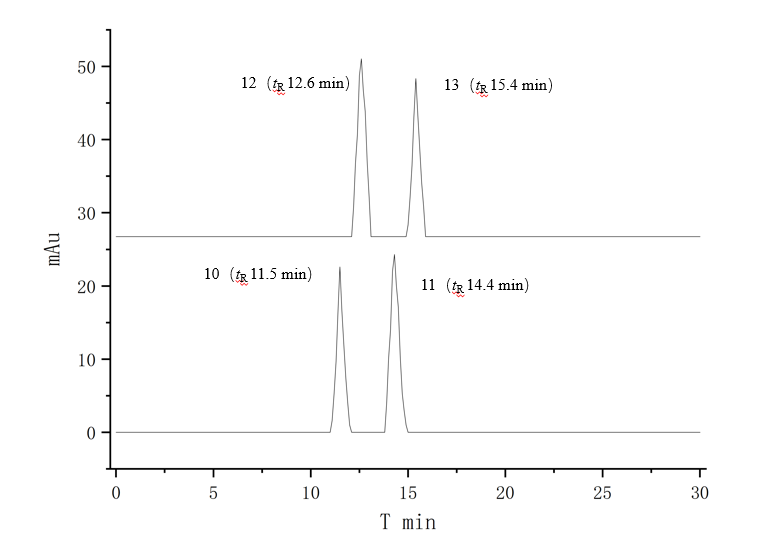


Figure S19. HPLC spectra of 10/11 and 12/13
